# A nonparametric clustering stopping rule based on spatial median

**DOI:** 10.1080/02664763.2025.2557964

**Published:** 2025-09-20

**Authors:** Hend Gabr, Brian H. Willis, Mohammed Baragilly

**Affiliations:** a Department of Mathematics, Insurance and Statistics, Faculty of Business, Menoufia University, Menoufia, Egypt; b Alliance Manchester Business School, University of Manchester, Manchester, UK; c Institute of Applied Health Research, University of Birmingham, Birmingham, UK; d Department of Mathematics, Insurance and Applied Statistics, Helwan University, Cairo, Egypt; e Department of Inflammation and ageing, University of Birmingham, UK

**Keywords:** Cluster-analysis, spatial median, stopping rule, multivariate data

## Abstract

In this work, we introduce a nonparametric clustering stopping rule algorithm based on the spatial median. Our proposed method aims to achieve a balance between the homogeneity within the clusters and the heterogeneity between clusters. The proposed algorithm maximises the ratio of the variation between clusters and the variation within clusters while adjusting for the number of clusters and number of observations. The proposed algorithm is robust against distributional assumptions and the presence of outliers. Simulations were used to validate the algorithm. We further evaluated the stability and the efficacy of the proposed algorithm using three real-world datasets. Moreover, we compared the performance of our model with 13 other traditional algorithms for determining the number of clusters. We found that the proposed algorithm outperformed 11 of the algorithms considered for comparison in terms of clustering number determination. The finding demonstrates that the proposed method provides a reliable alternative to determine the number of clusters for multivariate data.

Highlights

- A nonparametric clustering stopping rule algorithm based on the spatial median.
- Considers balancing intra-cluster homogeneity and inter-cluster heterogeneity.
- Maximizes between-cluster to within-cluster variation.
- Accounts for the number of clusters and number of observations.
- Outperforms 11 out of 13 traditional algorithms in cluster analysis.

## 1. Introduction

The problem of determining the number of clusters in a dataset is a relevant problem across a wide variety of disciplines such as business [1], psychology [2], statistics [3], medicine [4], computer science [5], and engineering [6]. A clustering stopping rule (also called an index) focuses on computing some functions for each cluster solution and chooses the one that indicates the most distinct clustering. Many publications have introduced various parametric statistical algorithms for identifying groups of points in multidimensional spaces while considering within and between cluster variations depending on the classical parametric measures ‘mean’ [7,8]. The nonparametric measures like spatial median and spatial rank represent another approach to determine the optimal number of clusters in multivariate datasets [9-12].

In many statistical studies, particular attention is paid to the spatial median due to its straightforward computational approach and its robustness against outliers. The spatial median has a breakdown point of 50%, meaning that it can tolerate up to 50% contamination by outliers before giving arbitrarily incorrect results. In contrast, the mean (used in traditional centroid-based clustering indices) has a breakdown point of 0%, making it extremely sensitive to even a single outlier. Therefore, BWDM using the spatial median yields cluster evaluations that are robust to outlying observations and skewed distributions. Moreover, using the ranks instead of the original observations provides more information about how central each observation is and in which direction it is moving from the center. The term ‘spatial median’ is presented by [13], who examined its properties and showed that the empirical distribution of the spatial median is asymptotically normal. Another property of the spatial median is its uniqueness whenever the dimension is two or more ( p≥2) and can be extended it into Banach spaces [14]. Other properties of spatial median have been introduced by [15] who showed that although the spatial median is equivariant under orthogonal transformations, it is not equivariant under general non-singular transformation. Thus, one can use the corresponding transformation-retransformation spatial median that has been extensively discussed in [16,17]. For both the spherical and general elliptic model, the transformation retransformation spatial median is efficient and performs well. Another affine equivariant spatial median has been introduced by [18] who introduced this equivariant version of spatial median by estimating the location and shape parameters simultaneously based on the spatial signs. Another fast and monotonically convergent algorithm in order to compute the spatial median is proposed by [19].

In this study we introduce a clustering stopping rule algorithm based on the spatial median that considers the within and between distances in the multidimensional space. We propose an algorithm aiming at achieving the trade-off between the homogeneity within the clusters and the heterogeneity between clusters while controlling for number of clusters as well as number of observations within each cluster. Homogeneity within the clusters means that data within the same cluster should be as similar as possible such that the closer the observations are to each other within a cluster, the more cohesive and well-defined the cluster is. Conversely, the heterogeneity between clusters implies that data belonging to different clusters should be as different as possible such that the greater the distance between clusters, the more distinct clusters are. The rationale behind our method is that as the number of clusters increases, the within-cluster variation will decrease, and the between-cluster variation will initially increase. Thus, maximizing the ratio of the variation between clusters and the variation within clusters while adjusting for the number of clusters and number of observations achieves the balance between cluster separation and cohesion within clusters.

This method aligns with ideas behind common criteria like the Elbow method in clustering analysis, where the goal is to find a balance between too few and too many clusters [20].

We evaluated the stability and the efficacy of the proposed methods using both simulated and real-world datasets. Moreover, we compared the performance of our model with other traditional methods for determining the number of clusters.

## 2. Definitions

### 2.1. The sample univariate median:

Suppose that $x_{1},\ldots ,x_{\text{n}}$ is a sample from a univariate distribution *F* and let $x_{\left(1\right)}\leq x_{\left(2\right)}\leq \ldots \leq x_{\left(\text{n}\right)}$ be the order statistics of this sample, then the univariate median is defined as the $\frac{1}{2}$th quantile of the underlying distribution. Alternatively, we can use the univariate sign function that has been defined in [20] in order to get the univariate median value, where the numbers of observations on the left and right of x is $\left|\sum_{i=1}^{n}\mathrm{sign}(x_{i}-x)\right|$ which means that the univariate median is

(1) $$\begin{array}{r} \text{any }x\in R\text{ that satisfies}\left|\sum_{i=1}^{n}\mathrm{sign}(x-x_{i})\right|=0 \end{array}$$

Unlike the univariate median, the multivariate median has various versions and computational methods. For instance, L_1_ –median, L_2_ –median (spatial median), L_p_ –median, half-space median, simplicial depth median, marginal median and Oja median are well known types of the multivariate median (see [21] for a comprehensive review of multivariate medians).

### 2.2. The spatial median

Suppose that $x_{1},\ldots ,x_{\text{n}}\in R^{d}$ where *d* is the number of variables and *n* is the number of observations, considering the multivariate sign function that defined in [20] and replacing the absolute value |⋅| in univariate median above (1) with the Euclidean norm ||⋅|| then the spatial sample median can be written as

(2) $$\begin{array}{r} \text{any }x\in R^{d}\text{ that satisfie}\left||\text{s}\sum_{i=1}^{n}\mathrm{sign}(x-X_{i})\right||=0 \end{array}$$

## 3. Method

We propose a novel clustering stopping rule based on two functions of L_2_ Euclidean distance to the spatial median. The first one is the Average Between-Distances to the Medians (ABDM), where it is depending on calculating the average of L_2_ distances between the medians of each pair of groups, and the second one is the Average Within – Distances to the Median (AWDM), which considers the average of L_2_ distance of each individual observation to the spatial median of its group.

### 3.1. Between and within distances to median ratio (BWDM)

Suppose that $x_{1},\ldots ,x_{\text{n}}\in R^{d}$ is a *d* – dimensional dataset with sample size *n*, for some cluster solution with total number of clusters *K*, let $k_{i}$ denotes the size of the i-th cluster, and $SM_{i}$ is spatial median of the i-th cluster considering the spatial median function defined in equation (2), then the ABDM for multidimensional data can be defined as

(3) $$\begin{array}{rl} \text{ABDM}(K) & =\frac{1}{\left(\begin{array}{c} K\\ 2 \end{array}\right)\;}\sum_{\begin{array}{c} i\neq j\\ j>i \end{array}}^{K}||\text{S}{\text{M}}_{i}-\text{S}{\text{M}}_{j}||\\ & =\frac{1}{\left(\begin{array}{c} K\\ 2 \end{array}\right)\;}{\left(\sum_{\begin{array}{c} i\neq j\\ j>i \end{array}}^{K}\sum {\left(\text{S}{\text{M}}_{i}-\text{S}{\text{M}}_{j}\right)}^{2}\right)}^{1/2} \end{array}$$

where $\left(\begin{array}{c} K\\ 2 \end{array}\right)$ is the total number of the possible combinations of each pair of clusters, ||⋅|| is the Euclidean norm. It is clear that the total number of possible combinations of the clusters’ pairs is a monotonically increasing in the number of clusters *K*. Moreover, the difference between possible combinations in the solution (*k* = i) and solution (*k* = i-1) equals i-1. For instance, the total numbers of the possible combinations of each pair of clusters for *k* = 6 is 15 pairs, while it is 10 when the number of clusters *k* = 5, so the difference between them is 5 which is i-1 for i = 6. Thus, ABDM serves as a measure of between-cluster variation, where it gives the biggest value for the most distinct clustering. Consequently, we can initially suggest that choosing the optimal number of clusters should be in accordance with the highest value of ABDM.

Similarly, AWDM serves as a measure of within-cluster variation, where it considers the distance between each observation in the cluster and its spatial median. Accordingly, we can define AWDM as:

(4) $$\begin{array}{r} \text{AWDM}(K)=\frac{1}{n\;}\sum_{i=1}^{K}\sum_{k=1}^{k_{i}}||x_{\mathrm{ik}}-\text{S}{\text{M}}_{i}|| \end{array}$$

where $\sum_{i=1}^{K}k_{i}=n$ is the total sample size. So, for each clustering solution (*k*), we get the corresponding averages ABDM and AWDM in order to calculate the ratio BWDM.

From (3) and (4), we proposed a Between and Within Distances to Median (BWDM) index as a ratio of ABDM to AWDM after assuming a specific denominator for each of them to account for number of observations and number of clusters. The denominators were specified following the same procedure of computing Calinski–Harabasz (CH index) that has been introduced by [22], where they considered the denominator of the between-variation as (*k*-1), and the denominator of the within-variation is the sample size minus the number of clusters (*n*-*k*). They showed that their criterion is analogous to the F-statistic in univariate analysis, where the degree of freedom is the same. Moreover, they pointed out that their criterion has been already used by [23] as an F-test in a multivariate cluster analysis. Accordingly, our new criterion BWDM index of clustering assignments over a given number of clusters *K*, is computed as following:

(5) $$\begin{array}{r} \text{BWDM}(K)=\frac{\text{ABDM}(K)/(K-1)}{\text{AWDM}(K)/(n-K)} \end{array}$$

In order to estimate the number of clusters $\hat{K}$ based on the cluster-stopping rule BWDM, we should choose the value of *K* that maximizes the BWDM index value, such that:

(6) $$\begin{array}{r} \hat{K}=\arg\underset{K\in \{2,\ldots ,K_{\max}\}}{\max}\text{BWDM}(K) \end{array}$$

This method gives the highest value for the most distinct clustering, indicating strong homogeneity within the clusters and clear heterogeneity between clusters. Our proposed method is designed to mathematically capture both intra-cluster homogeneity (via AWDM) and inter-cluster heterogeneity (via ABDM). By dividing ABDM by *K*−1 and AWDM by *n*−*K*, BWDM mirrors the structure of the CH index, which serves as a normalized F-ratio. These terms act as natural weights derived from degrees of freedom, ensuring that comparisons across cluster solutions are fair regardless of sample size.

### 3.2. The BWDM algorithm

Computation of the BWDM statistic proceeds as follows:

For each cluster solution:

1. Calculate the average between-distances to the medians ABDM(K) by applying equation (3).
2. Calculate the average within-distances to the medians AWDM(K) by applying equation (4).
3. Calculate the ratio of the average between and within distances to the median BWDM(K) by applying equation (5).
4. Repeat steps (1) to (3) until *K* = *K*_max_.
5. Choose the number of clusters *K* that maximizes the ratio BWDM(K) as shown in equation (6).

It is important to note that our method does not use the spatial median as a distance measure per se. Instead, the spatial median serves as a robust location estimator for each cluster. We then compute Euclidean distances of data points to this spatial median, which is conceptually similar to traditional clustering methods that compute distances to a central point (e.g. the mean in *k*-means or the medoid in *k*-medoids). This approach is well-aligned with robust clustering practices, where using robust central points enhances resistance to outliers and skewed distributions. Therefore, there is no paradox: the spatial median defines a robust center, and distances are calculated from this center using a standard distance metric.

### 3.3 Theoretical properties: optimality and convergence

Let Suppose that $x_{1},\ldots ,x_{\text{n}}\subset R^{d}$ be a dataset generated from a mixture of ${\text{K}}^{*}$ distinct, well-separated clusters, each with finite second moments and unique spatial medians. Let BWDM(K)be the clustering index defined in (5). Then, as the sample size *n* → ∞, the value of BWDM(K) converges in probability to a maximum at the true number of clusters $K={\text{K}}^{*}$. That is, $\underset{n\to \infty }{\lim}P\left[\text{arg}\underset{k\in \{2,\ldots ,K_{\max}\}}{\max}\text{BWDM}(K)=K^{*}\right]=1$

***Proof***: *Assumptions:*

1. The data are drawn from a mixture of K* mutually exclusive populations $X^{\left(1\right)}$, … , $X^{\left(K^{*}\right)}$ }, each with a continuous distribution and a unique spatial median $\mu^{\left(i\right)}\epsilon$ $R^{d}$.
2. Clusters are well-separated, i.e. $∥\mu^{\left(i\right)}-\mu^{\left(j\right)}∥\geq \delta >0\text{for all}i\neq j$
3. The clustering algorithm assigns observations correctly to clusters as n→∞ (i.e. consistent clustering).
4. The spatial median is a consistent estimator: $SM_{i}\overset{p}{\to }$ $\mu^{\left(i\right)}$ as $n_{i}\to \infty$.

Step 1: Convergence of Between-Cluster Distance (ABDM).

By the consistency of the spatial median and the Law of Large Numbers:

(7) $$\begin{array}{r} \text{S}{\text{M}}_{i}\overset{p}{\to }\mu^{\left(i\right)}\text{and}||\text{S}{\text{M}}_{i}-\text{ S}{\text{M}}_{j}||\overset{p}{\to }||M_{i}-\;M_{j}|| \end{array}$$

Therefore,

(8) $$\begin{array}{r} \text{ABDM}(\text{K})\overset{p}{\to }\frac{1}{\left(\begin{array}{c} k\\ 2 \end{array}\right)}\sum_{i<j}∥M_{i}-\;M_{j}∥ \end{array}$$

This value is maximized at $K={\text{K}}^{*}$. For $K>{\text{K}}^{*}$, at least one cluster is split, reducing pairwise distances. Thus, ABDM is asymptotically maximized at $K={\text{K}}^{*}$.

Step 2: Convergence of Within-Cluster Distance (AWDM).

Within each cluster $X^{\left(i\right)}$, let $f_{i}(x)$ be the density. The spatial median minimizes expected distance: $\mu^{\left(i\right)}=\arg\underset{\mu }{\min}E_{f_{i}}[∥x-\mu ∥]$

By the Strong Law of Large Numbers:

(9) $$\begin{array}{r} \frac{1}{n_{i}}\sum_{X_{\mathrm{ik}}\in X^{\left(i\right)}}∥x_{\mathrm{ik}}-\;SM_{i}∥\overset{p}{\to }E_{f_{i}}[∥x-\mu^{\left(i\right)}∥] \end{array}$$

Therefore:

(10) $$\begin{array}{r} \text{AWDM}(\text{K})\overset{p}{\to }\frac{1}{n}\sum_{i=1}^{K}n_{i}E_{f_{i}}[∥\text{x}-\mu^{\left(i\right)}∥] \end{array}$$

This is minimized at $K={\text{K}}^{*}$. Splitting increases AWDM due to unstable medians.

Step 3: Convergence of BWDM.

Since ABDM and AWDM both converge:

(11) $$\begin{array}{r} \text{BWDM}(\text{K})=\frac{\text{ABDM}(k)/k-1}{\text{AWDM}(k)/n-k}\;\overset{p}{\to }\text{BWD}{\text{M}}^{*}(k) \end{array}$$

This ratio is maximized at $K={\text{K}}^{*}$, hence: $\arg\underset{k}{\max}\mathrm{BWDM}(K)\overset{p}{\to }K^{*}$ which completes the proof.

## 4. Numerical examples

In this section we evaluate the performance of our proposed clustering stopping rule algorithm using three simulated datasets and three well-known real-world datasets.

### 4.1. Simulated data examples

In these examples, we applied a series of simulated cases to assess the efficiency of the proposed algorithm. Three models have been simulated from a bivariate normal distribution while varying the mixture probabilities and considering different numbers of true clusters.

#### 4.1.1. Simulated data 1

**Figure 1.**
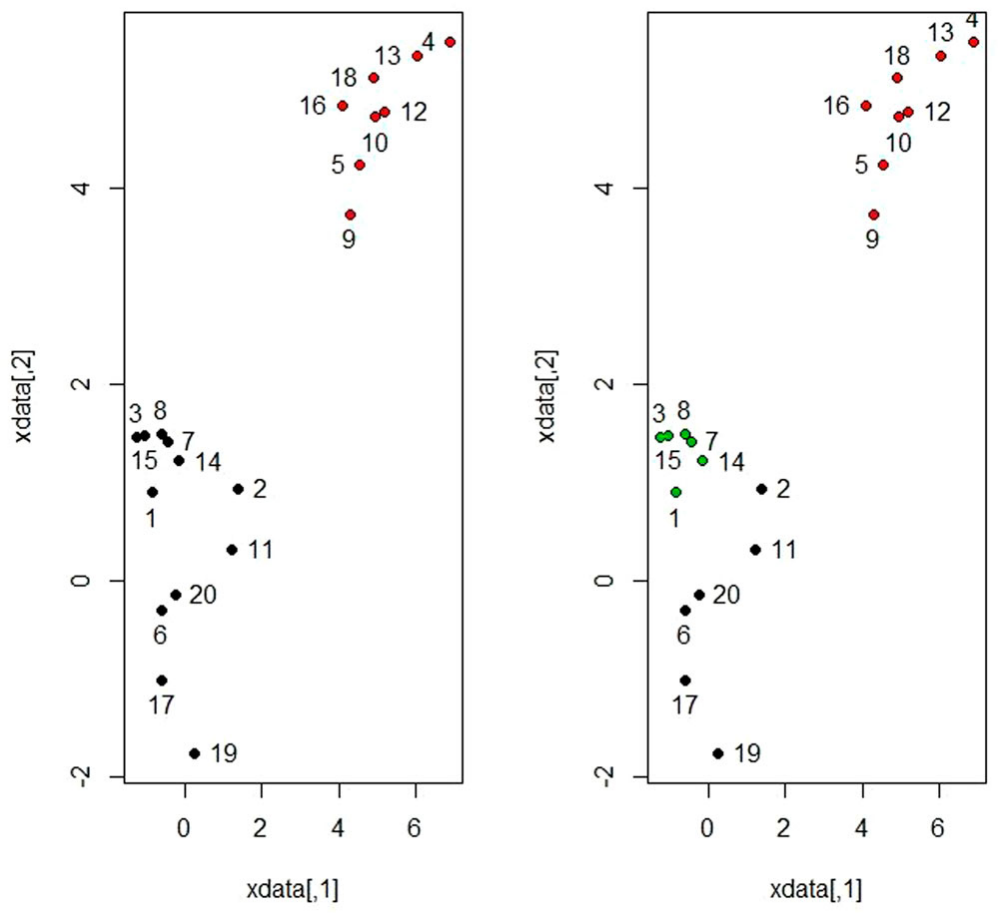
Scatterplot for a mixture of three groups for simulated data 1 assuming 2 and 3 clusters.

**Figure 2.**
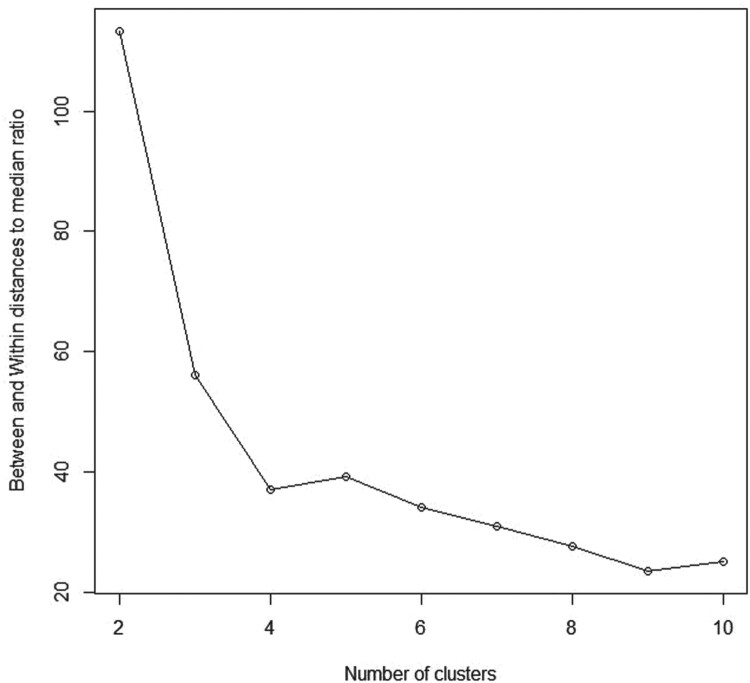
Between and within distances to median ratio (BWDM) curve for simulated data 1.

First, we generated data consisting of two groups from a bivariate mixture normal with mixture probabilities ( $p_{1},p_{2}$) such that

(12) $$\begin{array}{r} \;{\text{x}}_{1},\ldots ,\;{\text{x}}_{\text{n}}\;∼\;p_{1}N_{2}(\mu_{1},\;\Sigma )+\;p_{2}\;N_{2}(\mu_{2},\;\Sigma ) \end{array}$$

where $p_{1}=0.7$, $p_{2}=0.3$, $N_{2}(\mu_{1},\Sigma )$ and $N_{2}(\mu_{2},\Sigma )$ denote a bivariate Gaussian with means $\mu_{1}=\left(\begin{array}{c} 0\\ 0 \end{array}\right),\;\mu_{2}=\left(\begin{array}{c} 5\\ 5 \end{array}\right)$ and a covariance matrix Σ  = I. Second, we intentionally assumed that we have three groups to explore how the proposed method would respond under this scenario. Figure 1 shows the scatter plot for a mixture of two groups from bivariate normal distribution considering the two cases *K* = 2 and *K* = 3. The BWDM(K=2)=113.38, and BWDM(K=3)=56.13, which suggests, depending on BWDM criterion, that $\hat{K}=2$ results in greater cluster separation than $\hat{K}=3$. In order to check the stability of BWDM over other potential numbers of clusters, we assumed that the maximum number of clusters allowed (*K*_max_) equals 10 (as frequently used in literature). As shown in Table 1 $\hat{K}=2$ maximizes the BWDM index. Figure 2 shows the BWDM curve for the assumed model, at *K*_max_ = 10, with the maximum point of BWDM(K) occurs at $\hat{K}=2$, which confirms the stability of our method.

**Table 1.** Between and within distances to median ratio (BWDM).

*K*	2	3	4	5	6	7	8	9	10
*Simulated data*									
Simulated data1	113.38	56.13	37.10	39.33	34.20	30.99	27.58	23.48	25.10
Simulated data2	61.03	64.60	49.58	36.81	30.60	23.82	25.93	29.86	28.14
Simulated data3	1554.70	1296.54	1940.96	1403.62	1149.28	1010.36	910.49	781.04	767.38
*Real-world datasets*									
Real data 1	1516.09	767.43	642.35	581.33	533.80	519.28	473.76	430.85	470.21
Real data 2	353.53	171.58	145.52	122.62	101.98	89.56	75.44	69.41	62.30
Real data 3	684.95	377.18	241.28	196.46	188.34	150.59	127.31	112.85	108.59

**Figure 3.**
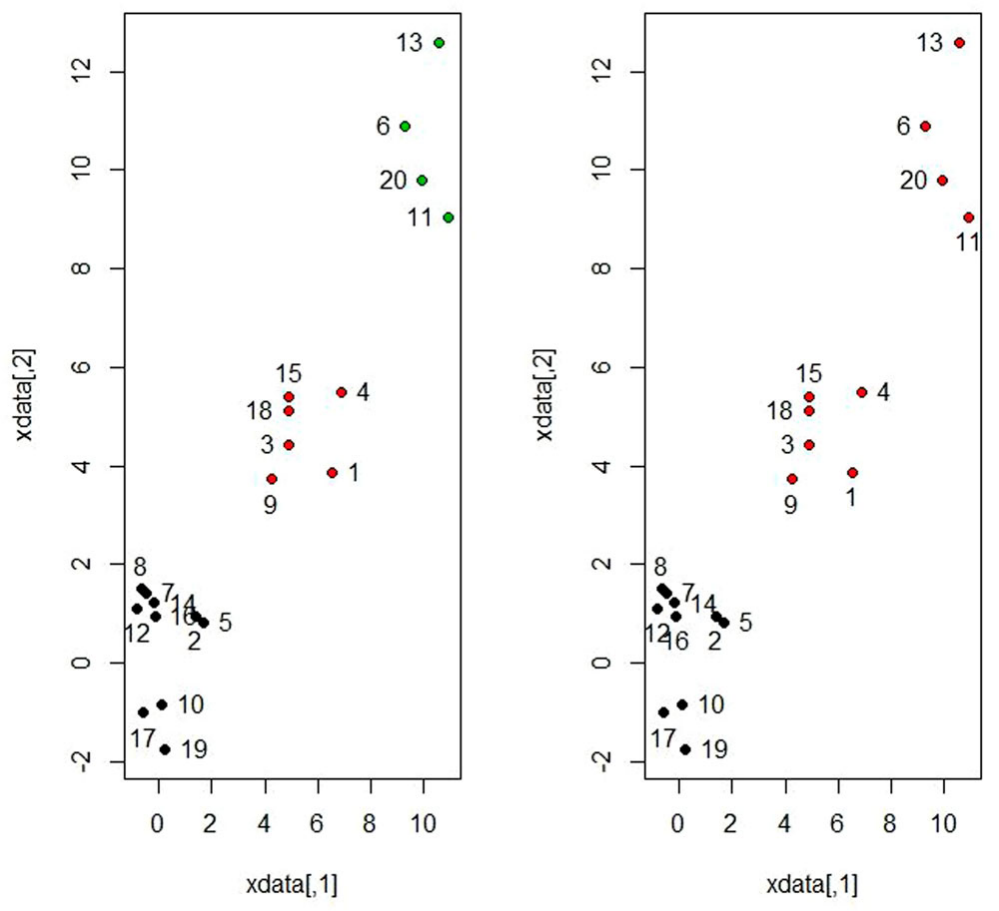
Scatterplot for a mixture of three groups for simulated data 2 assuming 2 and 3 clusters.

**Figure 4.**
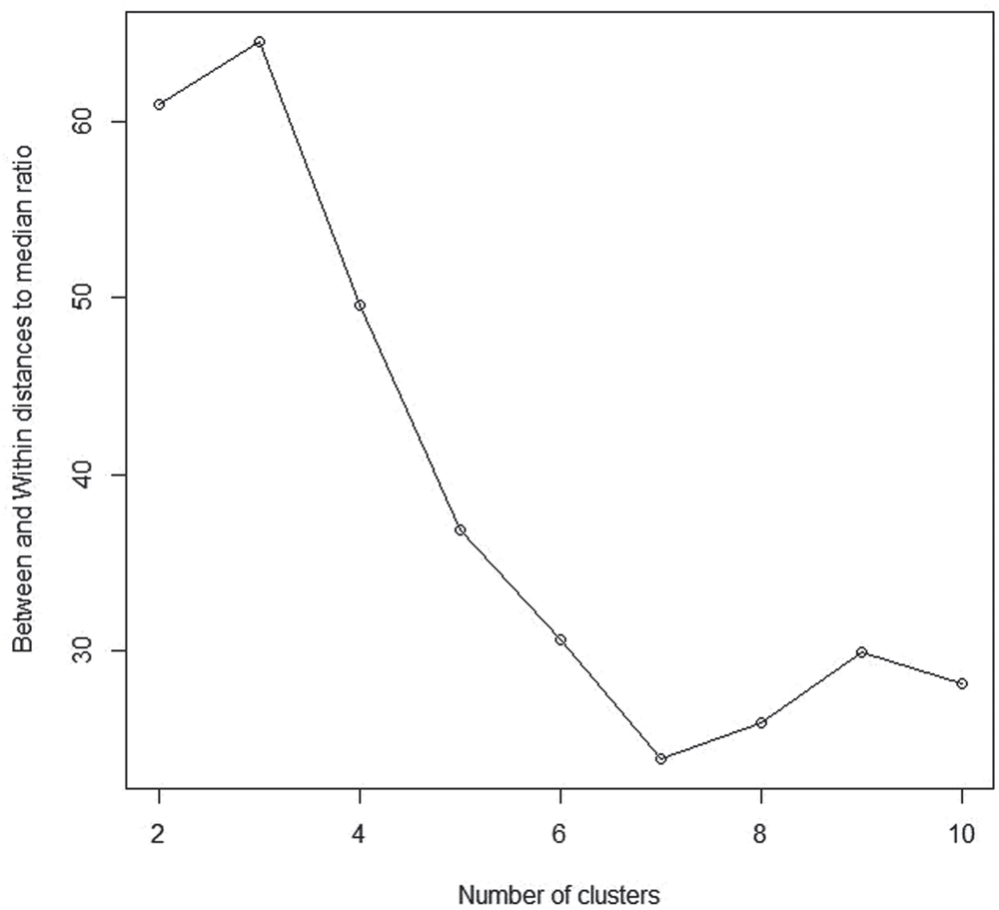
Between and within distances to median ratio (BWDM) curve for simulated data 2.

#### 4.1.2. Simulated data 2

In this scenario we generated data consists of three groups with mixture probabilities ( $p_{1},p_{2},p_{3}$) such that

(13) $$\begin{array}{r} \;{\text{x}}_{1},\ldots ,\;{\text{x}}_{\text{n}}\;∼\;p_{1}N_{2}(\mu_{1},\;\Sigma )+\;p_{2}\;N_{2}(\mu_{2},\;\Sigma )+\;p_{3}\;N_{2}(\mu_{3},\;\Sigma ) \end{array}$$

Where $p_{1}=p_{2}=0.3,\;p_{3}=0.4$, $N_{2}(\mu_{1},\;\Sigma ),\;N_{2}(\mu_{2},\;\Sigma )\;$and $N_{2}(\mu_{3},\;\Sigma )$ denote a bivariate Gaussian with means $\mu_{1}=$ $\left(\begin{array}{c} 0\\ 0 \end{array}\right),\;\mu_{2}=\left(\begin{array}{c} 5\\ 5 \end{array}\right),\;\mu_{3}=\left(\begin{array}{c} 10\\ 10 \end{array}\right)\;$and a covariance matrix Σ  = I. In contrast to the first scenario, we intentionally supposed that we have two groups while the true number of clusters is 3. Figure 3 shows the scatterplot for a mixture of two groups from bivariate normal distribution considering the two cases *K* = 2 and *K* = 3. As reported in Table 1, the BWDM(K=2)= 61.03, and BWDM(K=3)= 64.60, suggesting that $\hat{K}=3$ results in greater cluster separation than $\hat{K}=2$ according to our proposed BWDM criterion. Moreover, we again examined the BWDM criterion for different numbers of clusters where *K*_max_ = 10. The BWDM curve (Figure 4) indicates that $\hat{K}=3$ is a reasonable choice as since *K* = 3 represents indeed all the observations with more homogeneity within clusters and more heterogeneity between clusters compared to *K* = 2 as shown in Figure 3.

#### 4.1.3. Simulated data 3

In this data we consider a model consists of a mixture of four bivariate normal distributions where the weights are assumed to be equal ( $p_{i}=0.25;i=1,\ldots ,4$) such that:

(14) $$\begin{array}{r} \;{\text{x}}_{1},\ldots ,\;{\text{x}}_{\text{n}}\;∼\;p_{1}N_{2}(\mu_{1},\;\Sigma )+\;p_{2}\;N_{2}(\mu_{2},\;\Sigma )+\;p_{3}\;N_{2}(\mu_{3},\;\Sigma )+\;p_{4}\;N_{2}(\mu_{4},\;\Sigma ) \end{array}$$

Where $N_{2}(\mu_{1},\;\Sigma ),\;N_{2}(\mu_{2},\;\Sigma ),\;N_{2}(\mu_{3},\;\Sigma )\mathrm{and}\;N_{2}(\mu_{4},\;\Sigma )$ denote a bivariate Gaussian with means $\mu_{1}=$ $\left(\begin{array}{c} 0\\ 0 \end{array}\right),\;\mu_{2}=(4),\;\mu_{3}=\left(\begin{array}{c} -4\\ 4 \end{array}\right),\;$ and a covariance matrix Σ  = I.

**Figure 5.**
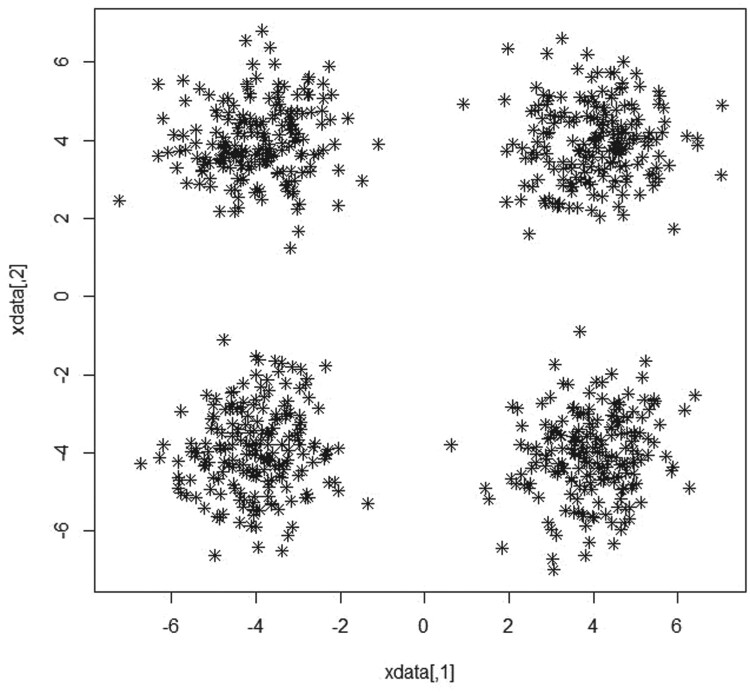
Scatterplot for a mixture of four bivariate normal distributions with equal weights and sample sizes.

**Figure 6.**
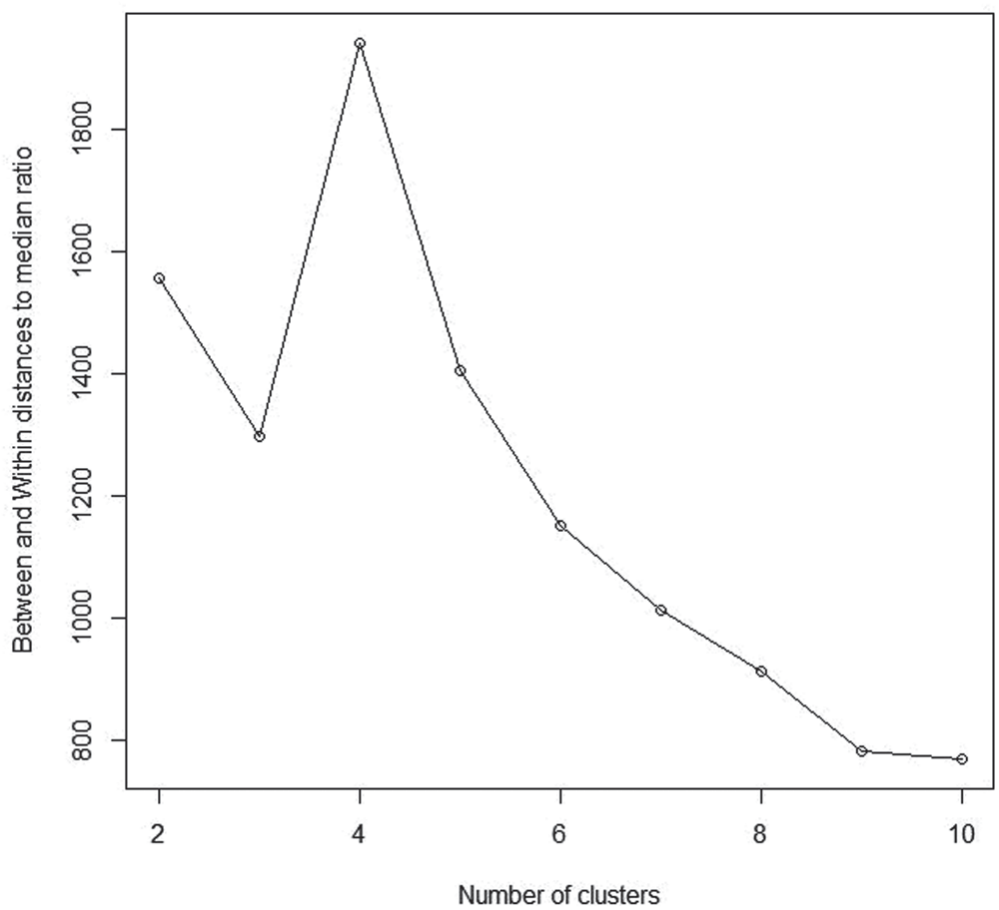
Between and within distances to median ratio (BWDM) curve for simulated data 3.

Similar to the previous scenarios, we intentionally assumed that we have a varied number of clusters different from the true number of four clusters with maximum number of clusters  = 10. Figure 5 shows the scatter plot for a mixture of four groups from bivariate normal distribution with equal probabilities. As shown in Table 1 and Figure 6, $\hat{K}=4$ maximizes the BWDM index which confirms the stability of our method.

### 4.2. Real-world datasets examples

In order to understand how well our algorithm works in practical situations, we evaluated the proposed clustering stopping rule algorithm using three well-known real datasets in statistics and data science including; Old Faithful data, Financial data, and Iris data.

#### 4.2.1. Real data 1: old faithful data

**Figure 7.**
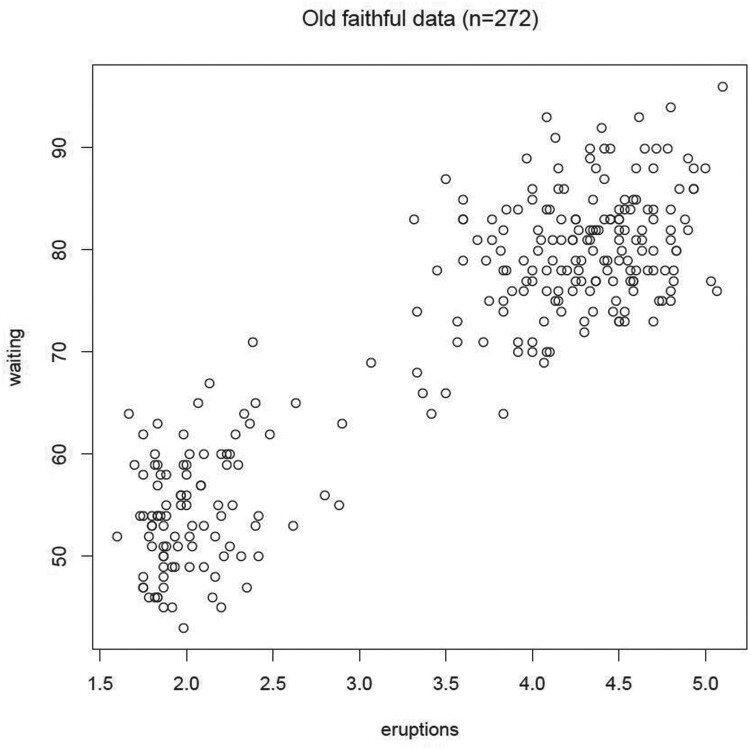
Scatter plot for Old Faithful Data.

**Figure 8.**
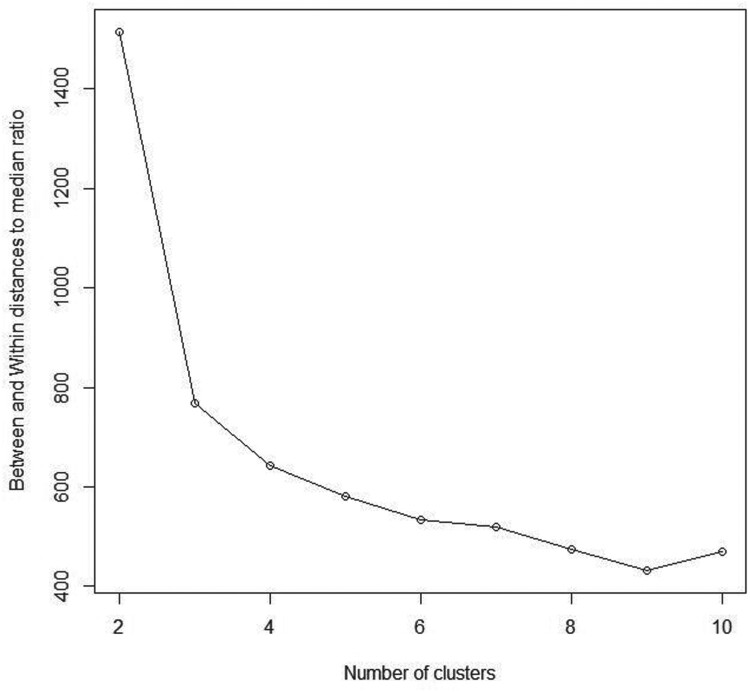
Between and within distances to median ratio (BWDM) curve for Old Faithful dataset.

This dataset includes two variables, the waiting time between eruptions, and the duration of eruptions in minutes for the old faithful geyser in Yellowstone National Park, Wyoming, USA [24]. As shown in the scatter plot of the faithful dataset (Figure 7), this dataset clearly consists of two clusters, the short and the long eruptions. Consistently, results of our proposed stopping rule algorithm presented in Figure 8 and Table 1 showed that $\hat{K}=2$ maximizes the BWDM index, suggesting that our proposed method performs well in this dataset.

#### 4.2.2. Real data 2: financial data

**Figure 9.**
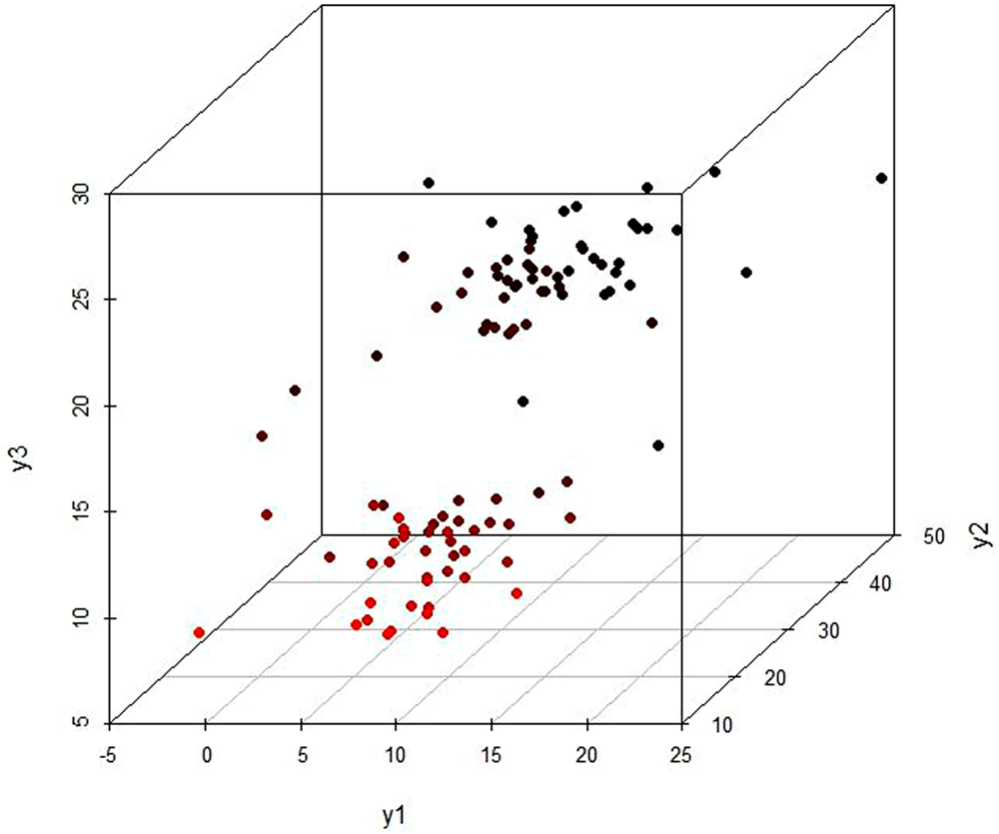
3D scatterplot for Financial data.

**Figure 10.**
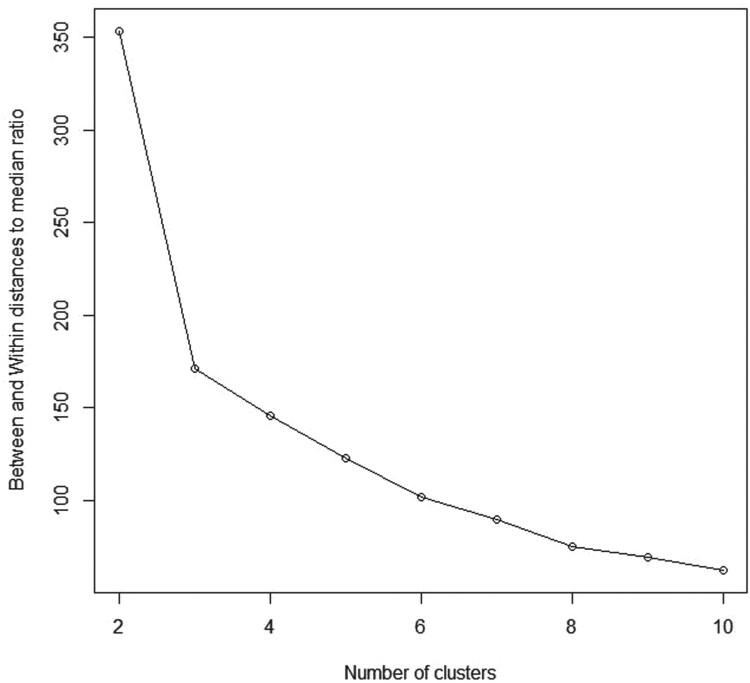
Between and within distances to median ratio (BWDM) curve for Financial data.

This dataset includes measurements of three variables that monitor the short term and medium term performance and medium term volatility of investment funds operating in Italy since April 1996 (Table A.16 of [25]). The financial data consists of two clusters representing two different kinds of funds; stock funds and balanced funds [26] presented by a 3D scatter plot (Figure 9). Our proposed method performs well in this dataset as well where $\hat{K}=2$ maximizes the BWDM index as shown in Figure 10 and Table 1.

#### 4.2.3. Real data 3: iris data

**Figure 11.**
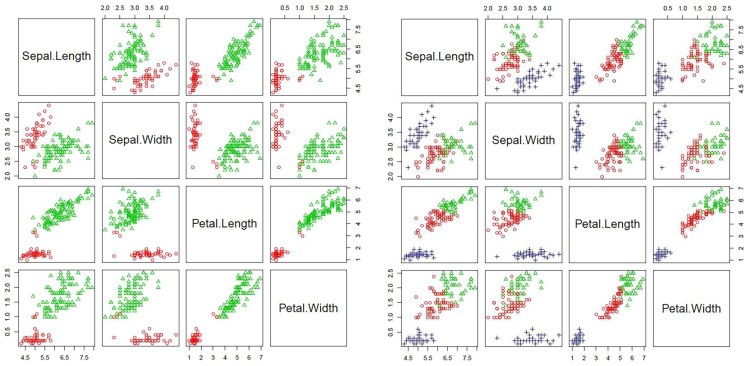
Scatter plot for Iris data assuming two and three clusters.

**Figure 12.**
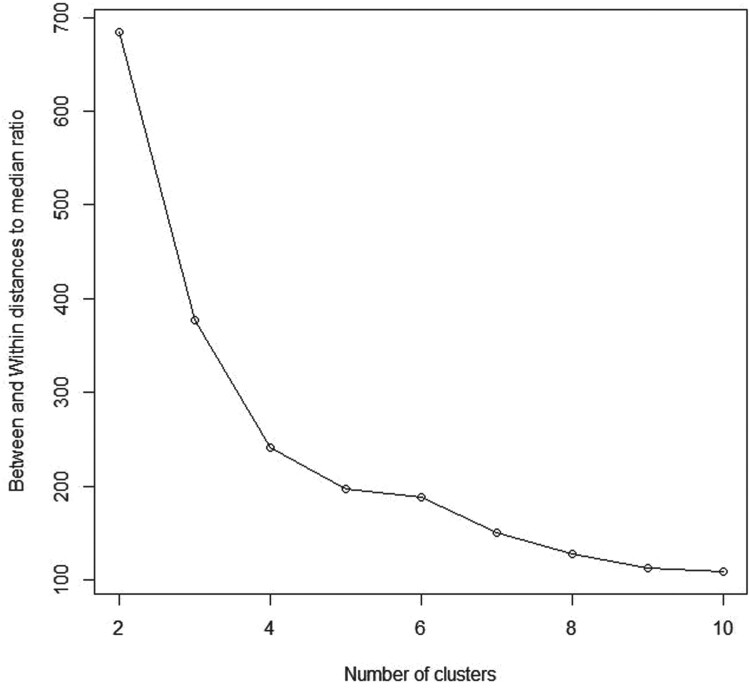
Between and within distances to median ratio (BWDM) curve for Iris data.

This dataset consists of four variables representing length and width of Sepal and Petal. Although these data contains three different types of irises (Setosa, Versicolour, and Virginica), most clustering techniques consider them as two groups since iris Virginica and iris Versicolour are not separable without the species information that Fisher used, as shown in Figure 11. Table 1 and Figure 12 showed that, our proposed algorithm continued to successfully determine the true number of clusters where $\hat{K}=2$ maximizes the BWDM index.

## 5. Comparison with other clustering algorithms

In this section we considered the three real data sets mentioned above to compare the performance of our method with other 13 clustering algorithms in order to assess its efficacy in determining the number of true clusters. The determined number of clusters for the algorithms used for comparison are presented in Table 2 along with our proposed algorithm (BWDM). These algorithms were applied to the Old Faithful data, the Financial data, and the Iris data. As shown in Table 2, five of the 13 algorithms (GMM (mclust), *K*-means, DBSCAN, KMD, and densityClust) did not identify the correct number of clusters for Old Faithful data. For Financial data, 6 algorithms (GMM (mclust), HDDC, DBSCAN, DDC, SNN, and densityClust) suggested wrong number of clusters while for the Iris data, only 5 algorithms were successful in identifying the correct number of clusters (GMM, PAM, DBSCAN, WSR, and densityClust). However, in addition to our algorithm, only two other algorithms were stable in identifying the correct number of clusters for all considered datasets (PAM and WSR) while most algorithms (GMM, *K*-means, HDDC, DBSCAN, KMD, DDC, SNN, and densityClust) identified the correct number of clusters for only one of the three datasets. Recent developments in clustering methods such as fuzzy and hybrid systems [27-31] have also aimed to improve robustness and adaptiveness. Compared to these, BWDM offers a simpler nonparametric alternative that does not require fuzzification parameters or probabilistic models.

**Table 2.** Comparison of different algorithm used to determine number of clusters for old faithful, financial, and iris datasets.

		Data		
Method	Criteria used to determine number of clusters	Old faithful data	Financial data	Iris data
BWDM	Considers the variation of between and within clusters.	2	2	2
GMM (mclust) [32]	Depends on Bayesian information criterion (BIC).	3	3	2
*K*-means [33]	Uses CH index.	10	2	3
HDDC [34]	Uses Bayesian information criterion (BIC).	2	3	3
MixtPPCA [35]	Uses Bayesian information criterion (BIC).	2	2	3
PAM [36]	Depends on the optimum average silhouette width.	2	2	2
DBSCAN [37]	Uses a density-based approach to identify regions of high density.	3	1	2
WSR [9]	A Weighted Spatial Ranks based approach.	2	2	2
KMD [38]	Depends on *K*-medoids algorithm.	3	2	3
FCM [39]	A fuzzy C-means clustering algorithm.	2	2	3
GG[40]	The Gath–Geva clustering algorithm.	2	2	3
DDC [41]	Distance density clustering.	2	3	3
SNN [42]	Clustering with shared nearest neighbor clustering.	2	3	3
densityClust [43]	Clustering by fast search and finding of density peaks.	1	1	2

## 6. Concluding remarks

This study presents a clustering stopping-rule algorithm for determining the optimal number of clusters, based on the spatial median. We proposed an algorithm that aims to balance the trade-off between intra-cluster homogeneity and inter-cluster heterogeneity. The main idea is maximizing the ratio of between-cluster to within-cluster variation, while accounting for both the number of clusters and observations. As a nonparametric approach, it is robust against distributional assumptions. In addition, as it is a spatial median-based approach it performs well under the presence of outliers. The algorithm is minimally sensitive to initialization because it evaluates each candidate value of *K* in the range *K* = 2 to *K* = *K*_max_. It does not rely on initial centroids or randomized initial partitions. For computing the spatial median, a standard convergence tolerance (e.g. 10−5) is used, but tests showed that values in the range of 10−4 to 10−7 produce stable results. Thus, the BWDM is less sensitive to initialization compared to methods like *k*-means or EM. Unlike EM-based clustering methods, our approach does not assume a mixture model or rely on latent variable estimation and thus sidesteps identifiability issues. However, in settings where data generation follows a known mixture model, EM methods may provide additional parametric interpretability, though at the cost of robustness to outliers.

This algorithm has been validated through simulations where we considered three different scenarios of data generated from bivariate normal distribution. We further assessed its stability and effectiveness using three real-world datasets: the Old Faithful data, Financial data, and Iris data. The proposed algorithm successfully identified the correct number of clusters for all datasets. In addition, we compared its performance to 13 traditional clustering algorithms, demonstrating that our approach outperformed 11 of these in determining the number of clusters which highlights the method's reliability for clustering in multivariate data.

Furthermore, we studied the theoretical properties in terms of optimality and consistency. Since BWDM is defined over a discrete set, optimization is done by evaluating the BWDM index for each integer *K*, thus eliminating the need for gradient-based optimization.

While the idea of balancing within- and between-cluster variation is not new, our method introduces several novel features. First, BWDM operationalizes this balance using spatial medians rather than means, enhancing robustness to outliers and skewed distributions. Second, the measure is fully nonparametric and distribution-free, unlike traditional methods such as the Calinski–Harabasz index, which implicitly rely on assumptions of Gaussianity and equal variances. Third, BWDM incorporates degrees of freedom to normalize the between- and within-cluster components, allowing for fair comparison across different cluster solutions regardless of sample size. These innovations make BWDM especially suitable for real-world data that often deviate from idealized statistical assumptions.

While BWDM offers robustness and simplicity, it does have limitations. First, the method assumes a roughly spherical cluster shape, which may reduce performance for elongated or irregular clusters. Second, the algorithm may be less effective in very high-dimensional settings due to the curse of dimensionality affecting distance metrics. Lastly, since BWDM relies on pairwise distances between medians, the computational cost increases quadratically with the number of clusters.

Several directions can be pursued to extend the algorithm's applicability. One potential area is the exploration of more complex clustering structures, such as overlapping clusters. A further possible extension is adapting the algorithm to handle more complex datasets such as functional datasets. This extension could lead to significant contributions in areas such as time series analysis and further practical values in various fields such as medical imaging, or environmental studies, where functional data types are prevalent.

## Data Availability

The data used to support the findings of the study are available in the public domain and are appropriately referenced in this article.
